# The importance of booster vaccination in the context of Omicron wave

**DOI:** 10.3389/fimmu.2022.977972

**Published:** 2022-09-08

**Authors:** Zichun Wei, Jiarui He, Conghui Wang, Jiaqi Bao, Taiyang Leng, Fei Chen

**Affiliations:** Department of Physiology, Jining Medical University, Jining, China

**Keywords:** SARS-CoV-2, Omicron, vaccine, booster, heterologous, homologous

## Abstract

Omicron (B.1.1.529) was first detected in a sample collected in Botswana on November 11, 2021, and has rapidly replaced Delta as the dominant global variant given the robust transmissibility. Moreover, it displays a lower virulence than other variants. However, the pathogenicity of Omicron appears to be underestimated in view of the increasing levels of herd immunity through natural infection or vaccination. Additionally, the volume of hospitalizations and deaths increase in proportion to the number of cases due to the high transmissibility of Omicron. Therefore, vaccination remains an important public health priority. Notably, a series of important mutations in the Omicron spike protein, especially in the receptor-binding domain and N-terminal domain, appears to be associated with immune escape capacity, reducing the willingness of people to receive vaccines. Herein, we provide an in-depth discussion to assess the effectiveness of the second and third vaccination against Omicron variant. On the one hand, the two-dose vaccination program adopted by many countries is insufficient to prevent Omicron infection given the mutations correlated with immune escape and the decline in vaccine efficacy over time. On the other hand, booster dose significantly increases the protective efficacy against Omicron infection. Most importantly, heterologous third dose vaccination induces a more robust immune response than homologous booster dose. Therefore, under the special background of this pandemic, there is an urgent need to accelerate the third dose of vaccination, especially providing better booster vaccination strategies, to combat emerging Omicron variant.

## 1 Introduction

**Figure 1:**
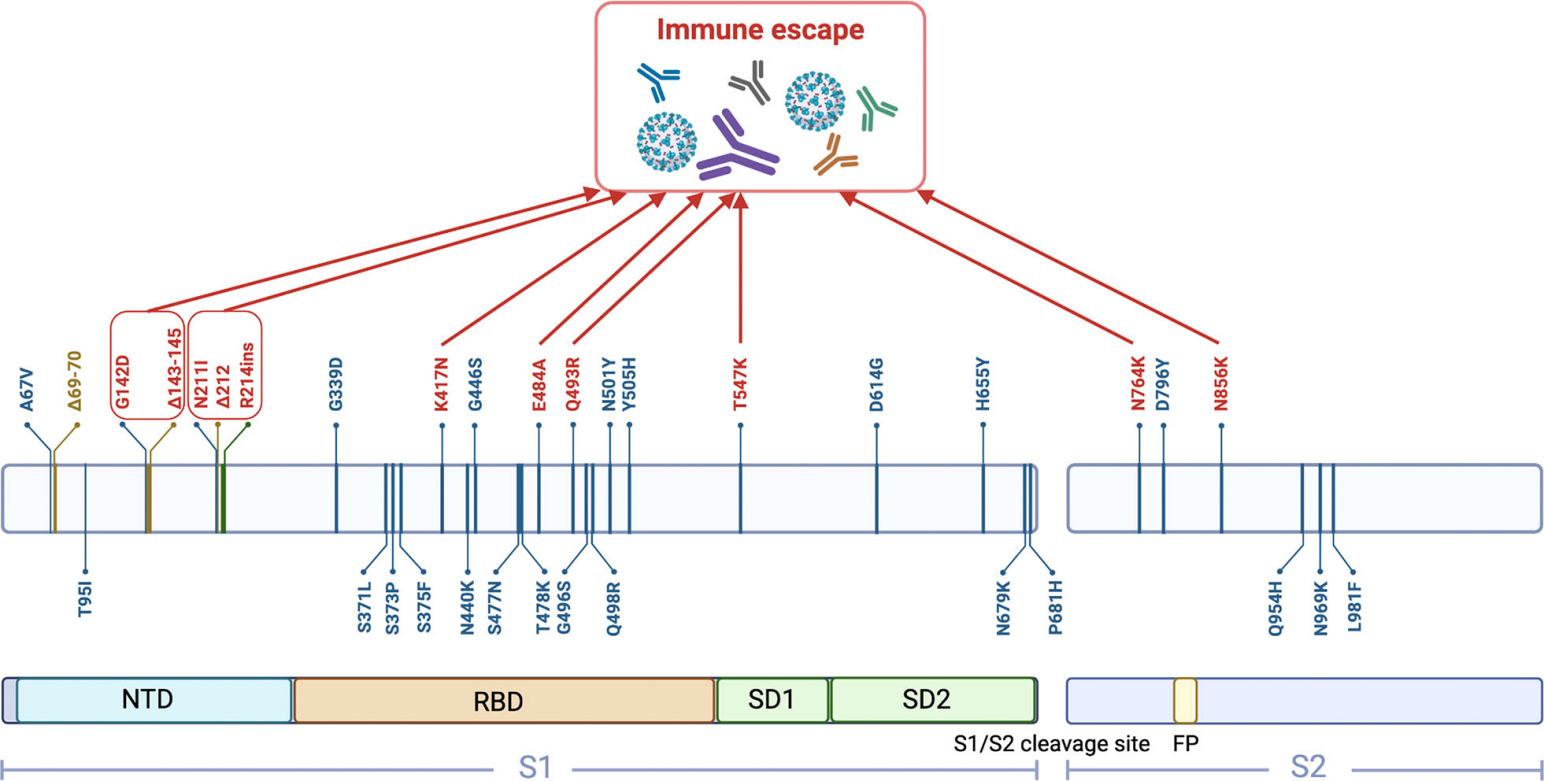
Schematic representation of 37 mutations in the BA.1 spike protein. (created with biorender.com). NTD, N-terminal domain; RBD, Receptor-binding domain; SD1, Subdomain 1; SD2, Subdomain 2; FP, Fusion peptide.

The coronavirus disease 2019 (COVID-19), caused by severe acute respiratory syndrome coronavirus 2 (SARS-CoV-2), continues to be prevalent worldwide. Like other RNA viruses, SARS-CoV-2 exhibits a drastically high rate of mutations because of the low fidelity of its RNA-dependent RNA polymerase, consequently acquiring genetic diversity and evolution (1). As the latest variant, Omicron (B.1.1.529) was first detected in a sample collected in Botswana on November 11, 2021, and was declared a variant of concern by the World Health Organization in the same month. Currently, this variant has been divided into five distinct lineages: BA.1, BA.2, BA.3, BA.4 and BA.5 (2). BA.1 is the originally dominating Omicron sub-lineage, making up more than 97% of Omicron sequences worldwide in November and December 2021, whereas BA.2 and BA.3 were rare (3). Thus, early studies of the Omicron were mainly based on this sub-lineage and revealed that it contains 37 mutations in the spike protein (4). Among them, 15 single mutations are located in the receptor-binding domain (RBD), with some changes and deletions in other genomic regions (**Figure 1**). It has been shown that the replication rate of the Omicron variant is over 70-fold higher in the bronchi compared to both wild-type SARS-CoV-2 and Delta variant (5). This may be because Omicron becomes less specialized in terms of its cellular tropism. Previous SARS-CoV-2 variants, such as Delta, only enter the host cells by binding angiotensin-converting enzyme 2 (ACE2) and activating fusion through cell-surface transmembrane serine protease 2 (TMPRSS2) (5, 6). However, the co-expression proportion of ACE2 and TMPRSS2 in the upper respiratory tract is exceedingly low (7), which may explain why previous SARS-CoV-2 tended to infect the lungs, rather than the upper respiratory tract. Conversely, BA.1 can enter host cells through TMPRSS2-dependent and non-dependent pathways. Because the number of cells with co-expression of ACE2 and cathepsins is seven times greater than those expressing both ACE2 and TMPRSS2 in the upper airway, the ubiquitous endosomal pathway can considerably increase the number of cell types that BA.1 can infect (6). Although knowledge of the effect of this rapid replication in the bronchi on pathogenicity is currently limited, higher infectious viral loads in the conducting airways are likely to increase the amount of infectious virus released during breathing or speaking, accelerating the transmission rate (8). Therefore, it is not difficult to understand why BA.1 has rapidly replaced Delta as the most predominant form of SARS-CoV-2 in the pandemic from November 2021 to January 2022. Then, BA.1 was replaced by BA.2 but with no increase in case numbers, and this was followed by a BA.4 and BA.5 infection surge between April and July 2022 (9).

There is growing evidence that Omicron causes milder symptoms than the earlier variants (10, 11). In fact, this could be attributed to the lower intrinsic virulence of Omicron (12), which does not efficiently spread through cell-cell fusion compared to other variants, thereby limiting viral infection in the lungs (13–15). Additionally, the Omicron variant has a 50–70% lower chance of hospitalization than the Delta variant according to a recent report (16). In line with this, Lewnard and colleagues found that hospital admissions with the Omicron were half of those with the Delta variant (17). Therefore, doubts have been raised about the necessity of vaccination (18, 19). However, the pathogenicity of Omicron might be underestimated due to increased levels of herd immunity *via* previous infections and vaccinations. For example, more than 70% of South Africans had detected antibodies against SARS-CoV-2 as of December 2021 (20). Moreover, hospitalizations also started rising rapidly after the notable increase in cases in view of the high transmissibility of the Omicron (21). In particular, the risk of severe disease remained high among comorbidities, immunocompromised patients as well as unvaccinated individuals (22). Malik et al. reported that COVID-19-related ICU admissions and deaths are also being driven by older adults (23). In addition, a massive surge of cases can not only place an overwhelming burden on the health system, medical resources and staff but may also maintain the viral infection chains in the populations, thereby enhancing the opportunity for SARS-CoV-2 mutations to become new variants (24). Therefore, efforts to improve vaccination coverage in countries, populations, and subgroups should remain a public health priority.

The protection from various vaccines against infection and the development of severe disease is waning slowly after two doses of the COVID-19 vaccine (25). Therefore, not only whether two doses of the vaccine can adequately protect against Omicron variant should be assessed but also alternative strategies beyond the two-dose regimen should be considered. Currently, booster programs have been recommended in more than 130 countries (26). However, a lack of awareness of the efficacy and safety of the third booster dose reduced public confidence in vaccination (27). Unlike homologous booster vaccination regimens which involve repeating the same vaccine multiple times, heterologous booster vaccination is a strategy that uses a different type of vaccine from the primary series of administered products (28). Its efficacy is associated with a great number of factors such as the order of vaccination (29). However, little is known about the immunogenicity and efficacy of different COVID-19 booster vaccination programs. Therefore, it is essential to evaluate the efficacy of the two-dose vaccination strategy and the booster strategy against Omicron, and to develop more effective booster vaccination regimens to combat the new threat posed by emerging Omicron variant.

### 1.1 Current risks of the two-dose vaccination strategy against Omicron

#### 1.1.1 Change in protective efficiency of vaccine over time

There has been a significant initial increase in antibody levels after the second dose of COVID-19 mRNA vaccine. However, these levels were reduced on average to 7% of peak levels at 6 months post-vaccination (30). This decline is expected as the rapid production of large amounts of antibodies is driven by short-lived plasma cells, which typically die within 1–2 weeks after antigen exposure. Moreover, the antibodies secreted by short-lived plasma cells decrease based on the specific half-life (approximately 21 days for immunoglobulin IgG) (31). Although low levels of IgA and IgG can be induced on the mucosal surface of the upper respiratory tract after intramuscular injection, this does not seem to provide effective protection against mucosal sites (32), because the IgA and IgG in the tracheal mucus disappear rapidly with the wane of serum antibody levels (31). Notably, a US study reported that 2,497 (55.9%) of 4,468 Omicron patients were fully vaccinated (33).

#### 1.1.2 Immune escape of Omicron

It is known that most approved or under development COVID-19 vaccines are designed to target the viral spike protein. Out of 47 vaccine candidates in clinical trials, about 35 vaccine candidates are based on the spike protein targeting through different technology platforms (34). So far, the potential fragments of spike protein for use as antigens include full-length spike protein, RBD, S1 subunit, N-terminal domain (NTD), and membrane fusion peptide (35). Omicron BA.1 hosts an unprecedented number of mutations in its spike protein compared with other variants, some of which are known to have functional consequences that lead to immune escape (**Figure 1**). As the binding site for neutralizing antibodies, RBD is an important domain of spike protein S1 subunit. The unique substitutions (T547K, N856K, and N764K in SD1 and S2) contribute to enhanced protomer–protomer and S1–S2 interactions. Therefore, the dominantly populated (61%) conformation for the spike protein trimer is in the closed state with all RBDs buried, resulting in conformational masking preventing antibody binding and neutralization at sites of receptor binding (36). This is analogous to human immunodeficiency virus type 1 evading antibody-mediated neutralization through envelope trimers (37). E484A, which is also present in BA.2, BA.3, BA.4 and BA.5 strains, results in overwhelmingly disruptive effects on many antibodies. Most of these damages are complementary to K417N, thereby making Omicron more likely to cause breakthrough infection (38, 39). Interestingly, the mutation at site 484 in Beta and Gamma is E484K, which can also escape the neutralization of antibodies, especially in combination with N501Y and K417N (40). The decreased effectiveness of vaccination is also associated with the Q493R mutation, which is caused by other neighboring mutations in conjunction with local conformational changes, resulting in greater resistance to antibodies through steric clashes (41). Notably, the mutations in NTD further alter the antigenic supersite. N211I and Δ212, as well as ins214EPE, alter the configuration of the loop, which includes residues 209–216 located near N4 loop. The substitution of G142D and Δ143–145 leads to a reconfiguring of N3 loop from a hairpin fold to a loose loop (42). Together, these two changes further alter the conformation of rings N4 and N5. BA.4 and BA.5 shared 12 RBD mutations with BA.1, some of which are associated with immune escape (e.g., N764K, E484A, and K417N), but it also has unique mutations (39). Therefore, it is important to understand its vaccine escape potential. BA.1 infection after vaccination mainly recalls wild-type-induced humoral immune response, which elicits antibodies that could be sufficient only to avoid severe infection with BA.1. However, most of these neutralizing antibodies are heavily escaped by L452R and F486V in BA.4 and BA.5, exhibiting poor neutralization breadth (43).

### 1.2 The safety and effectiveness of booster vaccination

Given that vaccine effectiveness rapidly wanes after two doses of vaccination, many countries have implemented booster dose programs to address the threat posed by Omicron. However, in the world, 61.60% of people have completed primary series vaccination, whereas only 28.76% of people received a booster by Aug 2, 2022. Particularly, in some low- and middle-income countries such as India, 66.30% of their population have been fully vaccinated, while the percentage of people who received a booster has been only 6.52% (44). Therefore, there is an urgent need for in-depth studies and a comprehensive understanding of relevant information on booster safety and effectiveness in order to ensure vaccination coverage. To date, there was no significant difference in the incidence of adverse reactions between the second and booster doses in most clinical studies (45, 46). This is not only in adolescents (47), but also in the elderly, patients with cancer, and organ transplant recipients (45, 48, 49). One retrospective study showed that the proportion of adverse events, such as fatigue, lymphadenopathy, nausea, and diarrhea increased in individuals who received the third dose compared with the second dose (50). However, the adverse reactions were usually mild and transient, resolving within a few days after onset (51). Additionally, the result of adverse reactions after vaccination relied on retrospective self-reports from subjects, so some memory bias might have occurred, especially considering the relatively long interval between the second and the booster vaccination. It is worth mentioning that the incidence of some serious adverse events such as myocarditis, is lower after the booster dose than after the second dose (52, 53).

Many experiments on the effects of different boosters have revealed that booster vaccination can provide good protection (54–56). The COV-Boost trial looked at using seven different vaccines as boosters after two doses of AstraZeneca or Pfizer: AstraZeneca, CureVac, Johnson & Johnson (Janssen), Moderna, Novavax, Pfizer and Valneva (57). Most vaccines have been found to boost immune responses, with the exception of CureVac, which was withdrawn. Additionally, neutralization efficiency of the BNT162b2 booster against the Omicron variant was 100-fold higher than the second dose (58). Given that there is a vast number of mutations in spike protein, particularly in RBD and NTD, Omicron variant has a stronger immune escape ability than previous variants. However, one study reported that Omicron and Delta variants respectively exhibited a 22.88-fold and 2.05-fold decrease in neutralizing antibody titers than ancestral D614G after the second dose of vaccine. After the third dose of vaccine, the neutralizing titers against the Omicron variant were only 3.28-fold reduced relative to D614G, which was less than the 4.28-fold reduction for the Delta variant (59). Although this experiment has further shown that booster vaccination could significantly increase neutralizing antibody titers, especially against the Omicron variant, this result should be viewed with caution. Particularly, in many neutralization trials, only this study reported this surprising result (60–62). Furthermore, Cheng et al. reported that low neutralizing antibody responses against Omicron BA.1 have been observed in individuals who received the booster of CoronaVac, leading to speculation that it would not provide effective protection against symptomatic disease caused by Omicron (63). However, moderate to high levels of protection against severe disease with three doses of CoronaVac vaccine were observed for more than two months in a clinical trial (64). This illustrates a gap in understanding the relevance of protection against severe disease, with a decoupling between measured neutralizing antibodies and clinical protection. Current clinical data indicate that booster vaccination provides better protection against the Omicron variant than primary series vaccination (64–70) (**Table 1**). Specifically, the efficacy of the two-dose vaccine regimen against symptomatic infection, severe infection and hospitalization declined over time, but effectiveness rapidly rebounded after the booster dose. In most studies, the efficacy of booster against symptomatic infection and severe infection is similar to or greater than that of two doses for the same time period after vaccination. Notably, it was higher than the second dose in preventing hospitalization. Although the booster vaccine effectiveness against hospitalization diminished over time, it was still greater than the maximum effectiveness of two doses (**Table 1**). Moreover, there was no significant difference in the protection of booster vaccination against BA.1 and BA.2 (65, 66). It is strongly recommended that people who have completed the second dose of COVID-19 vaccine should receive a third dose when eligible in view of the high infection rate during the Omicron wave.

**Table 1:** Vaccine effectiveness of primary series and booster dose against Omicron variant.

Omicron lineages	First and second dose	Booster	VE against symptomatic infection*		VE against severe infection*		VE against hospitalization*		Reference
			Primary	Booster	Primary	Booster	Primary	Booster	
Any Omicron	CoronaVac	CoronaVac	28.1% (14-59 days)	8.6% (8-59 days)	NP	NP	56.1% (14-59 days)	73.6% (8-59 days)	(64)
		BNT162b2		56.8% (8-59 days)	NP	NP		86.0% (8-59 days)
BA.1	BNT162b2	BNT162b2	-4.9% (median 268 days)	59.6% (median 42 days)	96.8% (median 268 days)	97.5% (median 42 days)	NP	NP	(65)
BA.2	BNT162b2	BNT162b2	-1.1% (median 270 days)	52.2% (median 43 days)	76.8% (median 270 days)	98.2% (median 43 days)	NP	NP
BA.1	mRNA-1273	mRNA-1273	-2.7%	56.5%	88.8%	100%	NP	NP
BA.2	mRNA-1273	mRNA-1273	-7.3%	52.9%	84.8%	100%	NP	NP
Any Omicron	BNT162b2	BNT162b2	-0.2%	54.0%	73.5%	92.5%	NP	NP
	mRNA-1273	mRNA-1273	2.2%	61.3%	66.3%	82.7%	NP	NP
Any Omicron	BNT162b2	BNT162b2	47.8% (1-3 months) 16.3% (4-6 months) -9.0% (≥7 months)	55.5% (2-3 weeks) 21.9% (≥14 weeks)	70.4% (1-6 months) 77.5% (≥7 months)	90.9% (1-6 weeks) 90.1% (≥7 weeks)	NP	NP	(66)
	mRNA-1273	mRNA-1273	43.2% (1-3 months) 18.7% (4-6 months) -13.7% (≥7 months)	53.7% (2-3 weeks) 34.9% (≥6 weeks)	87.1% (1-6 months) 68.4% (≥7 months)	81.8% (1-6 weeks) 100% (≥7 weeks)	NP	NP
BA.1	BNT162b2	BNT162b2	46.6% (1-3 months) 8.8% (4-6 months) -17.8% (≥7 months)	59.9% (<1 month) 40.5% (≥1 month)	NP	NP	NP	NP
	mRNA-1273	mRNA-1273	71.0% (1-3 months) 31.3% (4-6 months) -10.2% (≥7 months)	51.5% (<1 month) 45.3% (≥1 month)	NP	NP	NP	NP
BA.2	BNT162b2	BNT162b2	51.7% (1-3 months) 12.4% (4-6 months) -12.1% (≥7 months)	43.7% (<1 month) 40.2% (≥1 month)	NP	NP	NP	NP
	mRNA-1273	mRNA-1273	35.9% (1-3 months) 9.9% (4-6 months) -20.4% (≥7 months)	39.4% (<1 month) 41.9% (≥1 month)	NP	NP	NP	NP
Any Omicron	Any mRNA vaccine	Any mRNA vaccine	NP	NP	69.0% (<2 months) 48.0% (4 months)	87.0% (<2 months) 66.0% (4 months)	71.0% (<2 months) 65.0% (2-3 months) 58.0% (4 months) 54.0% (≥5 months)	91.0% (<2 months) 88.0% (2-3 months) 78.0% (≥4 months)	(67)
BA.1 (89%) BA.2 (11%)	Ad26.COV2.S / BNT162b2 / mRNA-1273	Ad26.COV2.S / BNT162b2 / mRNA-1273	NP	NP	NP	NP	54.0% (14-150 days) 42.0% (>150 days)	80.0% (7-120 days) 65.0% (>120 days)	(68)
Any Omicron	ChAdOx1 nCoV-19 / Ad26.COV2.S / BNT162b2 / mRNA-1273	BNT162b2 / mRNA-1273	37.0% (0-50 days) 18.0% (100-150 days)	52.0% (0-50 days) 25.0% (100-150 days)	NP	NP	59%	87.0% (0-50 days) 66.0% (100-150 days)	(69)
Any Omicron	BNT162b2	BNT162b2	65.5% (2-4 weeks) 48.7% (5-9 weeks) 8.8% (≥25 weeks)	67.2% (2-4 weeks) 55.0% (5-9 weeks)	NP	NP	NP	NP	(70)
	BNT162b2	mRNA-1273		73.9% (2-4 weeks) 64.4% (5-9 weeks)	NP	NP	NP	NP
	ChAdOx1 nCoV-19	BNT162b2	48.9% (2-4 weeks) 33.7% (5-9 weeks) -2.7% (≥25 weeks)	62.4% (2-4 weeks) 52.9% (5-9 weeks)	NP	NP	NP	NP
	ChAdOx1 nCoV-19	mRNA-1273		70.1% (2-4 weeks) 60.9% (5-9 weeks)	NP	NP	NP	NP
	ChAdOx1 nCoV-19	ChAdOx1 nCoV-19		55.6% (2-4 weeks) 46.7% (5-9 weeks)	NP	NP	NP	NP
	mRNA-1273	mRNA-1273	75.1% (2-4 weeks) 14.9% (≥25 weeks)	66.3% (2-4 weeks)	NP	NP	NP	NP
	mRNA-1273	BNT162b2		64.9% (2-4 weeks)	NP	NP	NP	NP

VE, Vaccine effectiveness; NP, Not provided.

*VE was calculated as [1 − odds ratio] x 100% and equates to the reduction in disease occurrence for those who are vaccinated (i.e. a VE of 85% = an 85% reduction in disease occurrence among the vaccinated).

### 1.3 Booster vaccination strategy

Traditionally, immune response is induced by single-shot vaccines and repeated injections of the same vaccine (homologous regimens). Nevertheless, current public health problems may require a qualitatively and quantitatively enhanced immune response, which is difficult to achieve with traditional vaccination methods. In contrast, heterologous booster vaccination regimen is a promising approach to inducing combined humoral and cellular responses. The main benefit expected is broader, stronger, and/or longer-lasting immunity (29). In previous studies, heterologous booster vaccination has already been successfully deployed for the treatment of numerous conditions, including Ebola virus disease, human immunodeficiency viruses, influenza, tuberculosis, and hepatitis B (28, 71). Given the increasing global demand for vaccine boosters, different booster vaccination regimens are needed to improve flexibility in the event of vaccine shortages. Particularly, given that inactivated viral vaccines are widely used around the world, suitable booster vaccination strategies are likely to be highly beneficial for billions of people who have been given the inactivated viral vaccines. However, knowledge related to the immunogenicity and efficacy of homologous or heterologous booster vaccinations remains limited. Therefore, the effectiveness of different booster vaccination regimens is urgently needed to be evaluated to inform vaccine policies in countries using these vaccines.

**Table 2** summarizes the geometric mean titer (GMT) following homologous and heterologous vaccination against different COVID-19 variants in the current studies (63, 72–78). It is noteworthy that levels of neutralizing antibodies produced in populations receiving a heterologous booster after two doses of inactivated viral vaccines are higher than that of the homologous group. Cheng et al. reported that the GMT against Omicron BA.1 variant was only 8.9 after three doses of homologous inactivated viral vaccines, while the GMT induced by mRNA heterologous booster could reach 59.2, which was about 6.65 times higher than that in the homologous group (63). Other tested heterologous booster vaccination strategies also induced a more robust immune response against Omicron compared to the corresponding homologous booster vaccination strategies (77, 79). However, the potential mechanism for higher immunity of heterologous vaccination remains largely unclear. The improved immune response may be attributed to different platforms that induce protection from different immune-mediated pathways, increasing the intensity and breadth of immune response. Specifically, the inactivated viral vaccines contain additional viral proteins that potentially provide protection beyond anti-spike protein response (80), mRNA vaccines elicit high neutralizing and binding antibody titers, while adenovirus vector vaccines produce polyclonal antibodies after vaccination (81). Thus, the combination of inactivated viral vaccines containing SARS-CoV-2 nucleocapsid protein with other platform vaccines increases the level of neutralizing antibodies and facilitates the modulation of antibody responses. Moreover, robust T cell response is considered to play a vital role in the protection against COVID-19. New evidence suggests that T cell response in the group receiving heterologous regimens is higher than that in the group receiving homologous regimens (82). This is supported by the robust cellular immune responses dominated by cytotoxic T cells and Th1 CD4+ T cells observed in animal experiments following heterologous vaccination regimens (83). Other possible mechanisms may also help explain this phenomenon. A following robust response of naive cells through epigenetic reprogramming may be generated *via* well-trained innate cells, hematopoietic stem cells, progenitor cells and resident memory T cells when a heterologous vaccine is administered (81). In contrast, after homologous vaccination, prior immunity to vaccines tends to impair robust antigen presentation and generation of appropriate inflammatory signals for T cells, resulting in relatively lower efficiency in enhancing cellular immunity (84). However, not all data have consistently shown that the heterologous booster elicited better immunogenicity than the homologous booster. Tan et al. reported that the immunogenicity of homologous BNT162b2 booster was superior to heterologous Ad26.COV2.S booster at 14 days (77). Such a result may be attributed to differential kinetics of immune responses induced by different types of vaccine, as the peak antibody titers of the BNT162b2 booster appeared at week 2, while the Ad26.COV2.S booster showed peak antibody titers at week 4 or later (85). Heterologous Ad26.COV2.S booster showed higher immunogenicity than homologous BNT162b2 booster at 28 days (**Table 2**).

**Table 2:** Comparison of geometric mean titer between heterologous and homologous regimens.

First and second dose(Type of vaccine)	Developer (s)	Booster(Type of vaccine)	Developer (s)	Sampling time after booster (days)	Efficacy against SARS-CoV-2 variant			Reference
					WT	Delta	Omicron	
BBIBP-CorV (Inactivated viral vaccine)	Sinopharm + Beijing Institute of Biological Products Co. Ltd	BBIBP-CorV (Inactivated viral vaccine)	Sinopharm + Beijing Institute of Biological Products Co. Ltd	14	285.60	250.80	48.73	(72)
		ZF2001 (Protein subunit vaccine)	Anhui Zhifei Longcom Biopharmaceutical + Institute of Microbiology, Chinese Academy of Sciences		1436.00	1501.00	95.86	
		BBIBP-CorV (Inactivated viral vaccine)	Sinopharm + Beijing Institute of Biological Products Co. Ltd	28	414.20	294.90	47.69	
		ZF2001 (Protein subunit vaccine)	Anhui Zhifei Longcom Biopharmaceutical + Institute of Microbiology, Chinese Academy of Sciences		789.60	534.00	108.70	
		BBIBP-CorV (Inactivated viral vaccine)	Sinopharm + Beijing Institute of Biological Products Co. Ltd	14	574.00	NP	84.00	(73)
		ZF2001 (Protein subunit vaccine)	Anhui Zhifei Longcom Biopharmaceutical + Institute of Microbiology, Chinese Academy of Sciences		1619.00	NP	172.00	
BBIBP-CorV/CoronaVac (Inactivated viral vaccine)	Sinopharm + Beijing Institute of Biological Products/Sinovac	Ad5-nCoV-IM (Non replicating viral vector vaccine)	CanSino Biologics Inc. + Beijing Institute of Biotechnology	14	970.00	NP	261.00	(74)
		Ad5-nCoV-IH (Non replicating viral vector vaccine)			567.00	NP	320.00	
		ZF2001 (Protein subunit vaccine)	Anhui Zhifei Longcom Biopharmaceutical + Institute of Microbiology, Chinese Academy of Sciences		308.00	NP	86.00	
		CoronaVac (Inactivated viral vaccine)	Sinovac		139.00	NP	54.00	
		Ad5-nCoV-IM (Non replicating viral vector vaccine)	CanSino Biologics Inc. + Beijing Institute of Biotechnology	28	628.00	NP	180.00	
		Ad5-nCoV-IH (Non replicating viral vector vaccine)			874.00	NP	353.00	
		ZF2001 (Protein subunit vaccine)	Anhui Zhifei Longcom Biopharmaceutical + Institute of Microbiology, Chinese Academy of Sciences		210.00	NP	63.00	
		CoronaVac (Inactivated viral vaccine)	Sinovac		69.00	NP	25.00	
CoronaVac (Inactivated viral vaccine)	Sinovac	BBIBP-CorV (Inactivated viral vaccine)	Sinopharm + Beijing Institute of Biological Products Co. Ltd	28	NP	69.60	24.60	(75)
		AZD1222 (Non replicating viral vector vaccine)	University of Oxford + AstraZeneca		NP	1003.00	250.00	
		BNT162b2 (mRNA vaccine)	BioNTech + Fosun Pharma + Pfizer		NP	1285.00	277.00	
		mRNA-1273 (mRNA vaccine)	Moderna + NIAID		NP	2168.00	512.00	
		CoronaVac (Inactivated viral vaccine)	Sinovac	21-35	65.00	NP	8.90	(63)
		BNT162b2 (mRNA vaccine)	BioNTech + Fosun Pharma + Pfizer		305.50	NP	59.20	
		CoronaVac (Inactivated viral vaccine)	Sinovac	14	34.30	20.00	5.83	(76)
		BNT162b2 (mRNA vaccine)	BioNTech + Fosun Pharma + Pfizer	8	207.00	160.00	23.80	
BNT162b2 (mRNA vaccine)	BioNTech + Fosun Pharma + Pfizer	BNT162b2 (mRNA vaccine)		14	306.00	184.00	27.60	
		BNT162b2 (mRNA vaccine)		0	129.00	56.00	20.00	(77)
		Ad26.CoV2.S (Non replicating viral vector vaccine)	Janssen Pharmaceutical (Johnson & Johnson)		281.00	66.00	21.00	
		BNT162b2 (mRNA vaccine)	BioNTech + Fosun Pharma + Pfizer	14	7554.00	3040.00	1018.00	
		Ad26.CoV2.S (Non replicating viral vector vaccine)	Janssen Pharmaceutical (Johnson & Johnson)		1462.00	1009.00	591.00	
		BNT162b2 (mRNA vaccine)	BioNTech + Fosun Pharma + Pfizer	28	3986.00	1541.00	345.00	
		Ad26.CoV2.S (Non replicating viral vector vaccine)	Janssen Pharmaceutical (Johnson & Johnson)		3597.00	2148.00	859.00	
mRNA-1273 (mRNA vaccine)	Moderna + NIAID	BNT162b2 (mRNA vaccine)	BioNTech + Fosun Pharma + Pfizer	14	5986.00	5925.00	513.00	(78)
Ad26.COV2.S (Non replicating viral vector vaccine)	Janssen Pharmaceutical (Johnson & Johnson)			28	3268.00	2987.00	189.00	

WT, Wild-type; NIAID, National Institute of Allergy and Infectious Diseases; NP, Not provided.

The neutralizing antibody titer measured between two studies differed by a factor of 14 after injecting of homologous BNT162b2 booster for the same time. The reason for this difference is mainly associated with the age distributions in the two study populations, coupled with the large differences in antibody titers produced after SARS-CoV-2 vaccination with respect to age, as the median age was 34 years in the study by Tan et al. and 53 years in the study by Khong et al. (76, 77). Specifically, aging causes immune dysfunction and worse immunogenicity in older adults, which in turn leads to low protection following vaccination (86). The germinal center is an important site for the proliferation and maturation of B cells, but due to the impaired somatic hypermutation of the germinal center in the elderly, the number of B cells secreting antibodies is decreased (87). Moreover, B cell antibody affinity maturation decreases, resulting in a lack of class switch recombination to produce secondary isotypes (IgG, IgE, and IgA), thus, it fails to respond to vaccinations (88). Furthermore, antibody production is vaccine dose-dependent and correlated with individual RBD-binding IgG levels and neutralizing titers, while RBD IgG levels are significantly higher in young adults than in older individuals, suggesting that the latter might require higher dose or different adjuvants (89). In terms of cellular immunity, older adults show lower polyfunctional CD4+ and CD8+ T cells responses than younger adults, as the upregulation of thymosuppressive cytokines can lead to thymic involution with age (87). Dendritic cell homing and dendritic cell-induced T cell proliferation are also reduced with aging (90, 91). By the way, specific T cells can produce different cytokines and exhibit cytotoxic responses to vaccines, but are diminished in the elderly (91).

The difference in antibody titers between the two studies may also be related to sex. Generally, women develop stronger humoral immune responses than men after vaccination (92). This may be because estrogen or prolactin can bind to intracellular receptors, such as the estrogen receptors α and β, to induce B cell activation, thereby resulting in higher levels of immunoglobulins (93, 94). However, testosterone can suppress the immune system by upregulating anti-inflammatory cytokines such as interleukin-10 (93). In addition to sex hormones, X chromosome also influences the immune system. Since X-linked genes are determinants of immunocompetence, damaging mutations or polymorphisms in any X-linked gene can possibly have more adverse immune consequences in males (XY) compared to females (XX) (95). It is noteworthy that miRNAs can regulate the development and function of immune cells, and X chromosome contains approximately 80 miRNAs, while Y chromosome contains only two miRNAs (96). More importantly, X-chromosome-encoded miRNA-18 and -19 play a role in sex-based immune differences (97). Moreover, microbiota composition modulates local and systemic immune responses in a sex-specific manner (98).

Although booster shot helps restore immunity, the GMT after vaccination is still lower against Omicron as compared with other variants (**Table 2**). Some vaccines against Omicron are being developed at a rapid rate given the current dire and rapidly evolving epidemiological situation. These vaccines can induce high levels of neutralizing antibody titers against the Omicron (99–103) (**Table 3**). Additionally, vaccine efficacy appears to be dose-related, with higher doses eliciting stronger immune responses. The GMT induced by two doses of 10µg CircRNA ^RBD-Omicron^ is approximately 4-fold higher than that induced by two doses of 5µg (100). Notably, a report found that those who have received the COVID-19 vaccines currently in use had increased neutralizing antibodies against the Delta variant after Omicron infection, thus implying the Omicron-specific vaccines may confer broad-spectrum protection against other variants (104). Indeed, Omicron-specific vaccines have little ability to neutralize early variants (**Table 3**). More basic immunology and biotechnology research is required in pursuit of better and long-lasting vaccination strategies given the widespread of COVID-19 and the rapidly changing crisis.

**Table 3:** Efficacy of Omicron vaccine against SARS-CoV-2 variants.

Vaccine regimen	Dosage	Efficacy against SARS-CoV-2 variants		Reference
		Omicron	Other variants	
mRNA-1273 + control mRNA vaccine	5µg + 1µg	GMT: 1204 (BA.1), 1040 (BA.2)	NP	(99)
	0.25µg + 1µg	GMT: 321 (BA.1), 289 (BA.2)	NP	
mRNA-1273 + mRNA-1273.529 vaccine	5µg + 1µg	GMT: 23517 (BA.1), 19005 (BA.2)	NP	
	0.25µg + 1µg	GMT: 7883 (BA.1), 6344 (BA.2)	NP	
CircRNA ^RBD-Omicron^ + CircRNA ^RBD-Omicron^	5µg + 5µg	GMT: 47000	Failed to cross-protect against the Delta variant	(100)
	10µg + 10µg	GMT: 220000		
WT vaccine + WT vaccine	10µg + 10µg	NT_50_: 503	NT_50_: 6400 (D614G), 3000 (Beta), 4000 (Delta)	(101)
Omicron RBD-LNP vaccine + Omicron RBD-LNP vaccine	10µg + 10µg	NT_50_: 18600	Failed to neutralize other tested SARS-CoV-2 variants	
mRNA-Omicron + mRNA-Omicron	5µg + 5µg	FRNT_50_:1/60872	Exhibited low level of cross-reactive neutralization	(102)
S-encoding mRNA vaccine candidate + RBD-encoded mRNA vaccine boosters based on SARS-CoV-2 prototype	4µg + 4µg	NT_50_: 423	NP	(103)
S-encoding mRNA vaccine candidate + RBD-encoded mRNA vaccine boosters based on Beta	4µg + 4µg	NT_50_: 1202	NP	
S-encoding mRNA vaccine candidate + RBD-encoded mRNA vaccine boosters based on Delta	4µg + 4µg	NT_50_: 3073	NP	
S-encoding mRNA vaccine candidate + RBD-encoded mRNA vaccine boosters based on Beta-Delta	4µg + 4µg	NT_50_: 1548	NP	
S-encoding mRNA vaccine candidate + RBD-encoded mRNA vaccine boosters based on Omicron	4µg + 4µg	NT_50_: 7710	NP	

WT, Wild-type; RBD, Receptor-binding domain; LNP, Lipid nanoparticle, GMT, Geometric mean titer; NT_50_, 50% neutralization titer; FRNT_50_, 50% focus reduction neutralization test; NP, Not provided.

As a single vaccine combining multiple components, multivalent vaccines were used successfully against infectious disease pathogens such as rotavirus, human papillomavirus, and influenza virus (105–107). Thus, multiple studies have been conducted to develop multivalent vaccines against SARS-CoV-2. Compared with monovalent vaccines, multivalent SARS-CoV-2 vaccines not only induce better immunogenicity against Omicron but also provides more effective protection against other variants (108–111) (**Table 4**). Notably, mRNA-1273.214 vaccine which was prepared from ancestral Wuhan-Hu-1 and Omicron B.1.1.529 spike protein mRNAs can induce strong neutralizing antibody responses to Omicron BA.4 and BA.5 subvariants (110). Hence, multivalent SARS-CoV-2 vaccines development is considered to play a constructive role in defending against the constantly mutating virus. Notably, a study found that the HB02 + Beta + Delta + Omicron tetravalent vaccine had lower neutralizing antibody titers against most variants than the trivalent vaccine (108). This seems to suggest that the immunogenicity produced by multivalent vaccines is not only related to the number of polymerized antigens but also to the ratio of antigen during the preparation process. Thus, although several Omicron vaccines and multivalent SARS-CoV-2 vaccines have been approved for clinical trials (112–115), these routes still need to be studied, optimized and adapted in the future. What is certain, however, is that they will have a broad application prospect in preventing SARS-CoV-2 infection.

**Table 4:** Efficacy of multivalent vaccine against SARS-CoV-2 variants.

Type of vaccine	Vaccine	Detection time interval (days)	Efficacy against SARS-CoV-2 variants		Reference
			Omicron	Other variants	
Multivalent vaccine	HB02 + Delta	42	GMT: 378	GMT: 1425 (HB02), 571 (Beta), 1810 (Delta)	(108)
	HB02 + Omicron		GMT: 1847	GMT: 2313 (HB02), 741 (Beta), 759 (Delta)	
	HB02 + Delta + Omicron		GMT: 2072	GMT: 3271 (HB02), 1753 (Beta), 1544 (Delta)	
	HB02 + Beta + Delta + Omicron		GMT: 1901	GMT: 2184 (HB02), 1933 (Beta), 962 (Delta)	
Monovalent vaccine	BBIBP-CorV	15	GMT: 45	GMT: 460 (Prototype), 295 (Alpha), 267 (Beta), 275 (Delta)	(109)
Multivalent vaccine	NVSI-06-08		GMT: 367	GMT: 3797 (Prototype), 3263 (Alpha), 4002 (Beta), 2609 (Delta)	
Monovalent vaccine	mRNA-1273	28	GMT: 1473	GMT: 5649 (D614G)	(110)
Multivalent vaccine	mRNA-1273.214		GMT: 2372	GMT: 5977 (D614G)	
Monovalent vaccine	WT vaccine	NP	NT_50_: 1874	NT_50_: 20480 (D614G), 8570 (Beta), 9939 (Delta)	(111)
	Omicron vaccine		NT_50_: 6738	NT_50_: 13435 (D614G), 6205 (Beta), 4446 (Delta)	
Multivalent vaccine	Hybrid vaccine		NT_50_: 11776	NT_50_: 26957 (D614G), 13397 (Beta), 10889 (Delta)	

WT, Wild-type; GMT, Geometric mean titer; NT_50_, 50% neutralization titer; RBD, Receptor-binding domain; NP, Not provided.

## 2 Conclusion

The Omicron variant has a stronger immune escape ability than other variants, because it carries a large number of mutations, especially in RBD, which is the main target for neutralizing antibodies. Additionally, the antibodies induced by vaccines will decay over time. Both of these factors present a serious threat to the two-dose vaccination regimen, preventing it from providing adequate protection against the Omicron variant. Therefore, booster vaccination has become a topic of current interest. Although the rate of adverse events increased in individuals who received third dose of BNT162b2 or mRNA-1273 vaccine, these adverse reactions were only mild and transient, resolving within a few days. Furthermore, the prevalence of self-reported adverse reactions may be influenced by memory bias. Booster vaccination not only results in significantly increased neutralizing antibody titers against the Omicron variant but also provides stronger protection against symptomatic disease, especially in preventing hospitalization.

A heterologous booster shows higher vaccine effectiveness than a homologous booster, particularly in individuals who have been vaccinated twice with inactivated viral vaccines. Interestingly, homologous booster with BNT162b2 developed significantly higher antibody responses on day 14 than heterologous booster with Ad26.COV2.S, whereas the opposite effect was found on day 28. Such a result may be attributed to differential kinetics of immune responses induced by different types of vaccines. Notably, the efficacy of homologous BNT162b2 booster vaccination may be affected by individual differences, such as age or sex. Given that older people are one of the populations with the highest risk of infection, some countries recommend a fourth dose of the COVID-19 vaccine for them to improve protection against the Omicron variant. However, in low-income and middle-income countries, only a small percentage of people are vaccinated, with one dose being provided for their most vulnerable communities. Due to vaccine inequity and low vaccination coverage being major threats that undermine the global pandemic control efforts, it is of great importance to develop appropriate use criteria for the fourth dose of the COVID-19 vaccine. In addition, Omicron vaccines can generate strong neutralizing antibody responses against Omicron but have no neutralizing capacity against other variants, thereby revealing the need for more basic immunological studies to seek better and long-lasting vaccination strategies. In fact, the multivalent SARS-CoV-2 vaccines can not only improve immune response but can also effectively expand the scope of immunity. In summary, booster programs need to be implemented as soon as possible to manage the current pandemic and control infection rates in the post-pandemic world. Additionally, every individual should continue with strict vigilance on following infection control guidelines even if vaccinated in view of a severe surge of vaccine breakthrough infections.

## Author contributions

All authors contributed to the article and approved the submitted version.

## Funding

This work was supported by the Natural Science Foundation of Shandong Province (Grant No. ZR2020QC100) and the Innovation and Entrepreneurship Training Program for College Students of Jining Medical University (Grant No. cx2021062).

## Conflict of interest

The authors declare that the research was conducted in the absence of any commercial or financial relationships that could be construed as a potential conflict of interest.

## Publisher’s note

All claims expressed in this article are solely those of the authors and do not necessarily represent those of their affiliated organizations, or those of the publisher, the editors and the reviewers. Any product that may be evaluated in this article, or claim that may be made by its manufacturer, is not guaranteed or endorsed by the publisher.
